# Short-term effects of air pollution on respiratory diseases among young children in Wuhan city, China

**DOI:** 10.1007/s12519-022-00533-5

**Published:** 2022-03-25

**Authors:** Zeng-Hui Huang, Xing-Yuan Liu, Tong Zhao, Kui-Zhuang Jiao, Xu-Xi Ma, Zhan Ren, Yun-Fei Qiu, Jing-Ling Liao, Lu Ma

**Affiliations:** 1 School of Public Health, Wuhan University, No. 115 Donghu Road, Wuhan, 430071 Hubei China; 2 Wuhan Information Center of Health and Family Planning, Wuhan, China; 3 School of Environmental and Municipal Engineering, Xi’an University of Architecture and Technology, Xi’an, China; 4 Department of Nutrition and Food Hygiene, School of Public Health, Medical College, Wuhan University of Science and Technology, Wuhan, China

**Keywords:** Air pollution, Children, Hospitalization, Respiratory disease

## Abstract

**Background:**

The high risks for childhood respiratory diseases are associated with exposure to ambient air pollution. However, there are few studies that have explored the association between air pollution exposure and respiratory diseases among young children (particularly aged 0–2 years) based on the entire population in a megalopolis.

**Methods:**

Daily hospital admission records were obtained from 54 municipal hospitals in Wuhan city, China. We included all children (aged 0–2 years) hospitalized with respiratory diseases between January 2017 and December 2018. Individual air pollution exposure assessment was used in Land Use Regression model and inverse distance weighted. Case-crossover design and conditional logistic regression models were adopted to estimate the hospitalization risk associated with air pollutants.

**Results:**

We identified 62,425 hospitalizations due to respiratory diseases, of which 36,295 were pneumonia. Particulate matter with an aerodynamic diameter less than 2.5 μm (PM_2.5_) and nitrogen dioxide (NO_2_) were significantly associated with respiratory diseases and pneumonia. ORs of pneumonia were 1.0179 (95% CI 1.0097–1.0260) for PM_2.5_ and 1.0131 (95% CI 1.0042–1.0220) for NO_2_ at lag 0–7 days. Subgroup analysis suggested that NO_2_, Ozone (O_3_) and sulfur dioxide (SO_2_) only showed effects on pneumonia hospitalizations on male patients, but PM_2.5_ had effects on patients of both genders. Except O_3_, all pollutants were strongly associated with pneumonia in cold season. In addition, children who aged elder months and who were in central urban areas had a higher hospitalization risk.

**Conclusions:**

Air pollution is associated with higher hospitalization risk for respiratory diseases, especially pneumonia, among young children, and the risk is related to gender, month age, season and residential location.

**Supplementary Information:**

The online version contains supplementary material available at 10.1007/s12519-022-00533-5.

## Introduction

Ambient air pollution has become one of the most severe public health threats in the world. The World Health Organization estimated that 22% of global death and disability were attributable to environmental risk factors [1, 2]. Children are uniquely vulnerable and susceptible to air pollution, particularly in their earlier years and even during fetal development, owing to their immature lungs, brains, organs and immune systems. Children breath faster than adults, thereby taking in more air pollutants with more air [3]. Thus, early exposure to ambient air pollution may affect the normal growth of children, especially when their lungs are rapidly developing and therefore are more vulnerable to respiratory disease caused by air pollutants [4]. To date, respiratory diseases is still the predominant disease affecting children's health. Pneumonia is the leading cause of childhood death among respiratory diseases, which killed 808,694 children 0–5 years of age worldwide in 2017 (including 107,811 Chinese children), accounting for 15% of all deaths of children under 5 years [3].

Several studies have shown the effects of exposure to air pollutants on the respiratory disease of children [5–8]. These studies include cohort, cross-sectional, and time series studies, but the results were inconsistent in many aspects, such as the results of parameter estimation, gender and seasonal effects, and the effects of exposure to different pollutants. In addition to research design, subject selection, and regional differences, the common limitations of these studies contributed to this inconsistency. On one hand, the study population of these studies was obtained by specific sampling methods and may not represent the entire population, which may introduce selection bias. On the other hand, to examine the exposure effects of air pollutants to respiratory diseases, most of studies estimated individual exposure level using the air pollutants’ concentrations from the nearest fixed stations or the city's average concentrations of air pollutants, which may easily lead to a misclassification [9]. Therefore, a study based on the entire population of a large city area and assessing the individual exposure levels upon their precise location may be better for understanding the real impact of air pollutants on respiratory diseases.

Most previous studies have included children aged 0–5 or 0–18 years, and fewer studies have focused on young children aged less than two years [9]. In fact, children at this age are more vulnerable to air pollution because their immune system and lungs are immature and not fully developed [10, 11]. Thus, they are at high risk of respiratory diseases related to air pollution [12, 13]. Moreover, exposure during early life may cause adverse health outcomes in later life. A study demonstrated that prenatal exposure and postnatal exposure to air pollution were associated with developmental delays in children under 2 years of age, specifically at ages less than 1 year [14]. Several lines of evidence have shown that early life is a critical exposure window for ambient air pollution, with increasing the risks of diseases in respiratory and nervous system. Additionally, early life exposure to air pollution also is associated with reduced lung function and other adverse health outcomes in adulthood [5, 15].

The aim of the present study is to explore the association between ambient air pollutants and hospital admission for respiratory disease, including pneumonia among young children aged less than 2 years. This case crossover study was conducted based on the admission data of all hospitals in Wuhan, which is a megacity in central China. Considering the universal access to hospital health care and the availability of these records, the inclusion of almost the whole population can eliminate the sampling error as much as possible, and the case crossover design also can enable us to control the interference of confounding factors at the individual level. At the same time, the assessment of individual exposure concentrations allowed the impact of air pollution on the entire young children population in Wuhan to be accurately evaluated.

## Methods

### Research location and study population

**Fig. 1:**
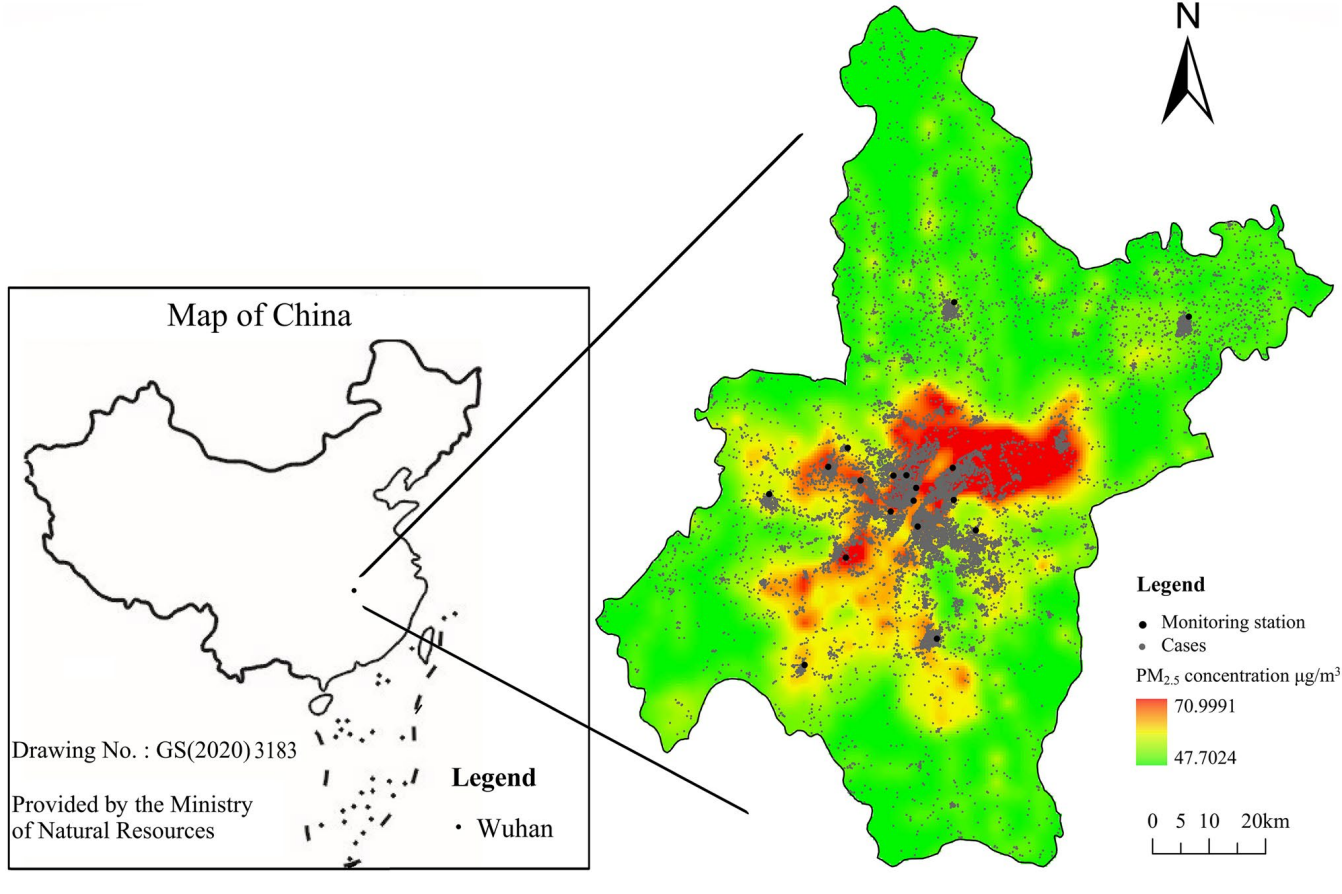
Location of air monitoring station and cases in Wuhan, China and the results of LUR model. This map is adapted from the standard map provided by Ministry of Natural Resources of China. LUR, land use regression; PM_2.5_, particles with aerodynamic diameter ≤ 2.5 μm. The map is obtained from Ministry of Natural Resources, China with the drawing no. GS(2020) 3183, which is permitted to be used publicly

With a land area of 8569.15 km^2^ and a population of about 11.2 million (Wuhan Statistical Y earbook-2019), Wuhan (29.58–31.22°N, 113.41–115.05°E) is the capital city of Hubei Province and is a megacity in central China (Fig. 1) [16].

Daily hospitalization data of 54 municipal hospitals in Wuhan were obtained from the Comprehensive Health Information statistics platform of Hubei Province, which includes basic information such as sex, age, residential address, date of hospitalization and discharge, disease diagnosis and number of hospital admission. The Wuhan Children's Hospital and the Maternal and Child Hospital of Hubei Province, which serve most of children patients of whole Wuhan city, are included among the 54 municipal hospitals. We included all infants aged 0–2 years admitted to hospitals with respiratory disease (International Classification of Diseases, 10th Revision: J00-J99) between January 2017 and December 2018 (*n* = 82,793). Patients whose residential address could not be geocoded or who came from locations other than Wuhan were excluded (*n* = 12,421). Patients who were recorded with wrong gender (*n* = 166) or who were not first hospital admission (*n* = 6,904) were excluded. Neonatal admissions (aged 0–30 days) were excluded because they were easily affected by perinatal environment (*n* = 877) [17]. Based on the above inclusion and exclusion criteria, a total of 62,425 hospital admission records were collected during this study period (Fig. S1).

### Study design

The case-crossover design is proposed for assessing the short-term effects of environmental exposures on the risk of acute events [18, 19]. Because each patient serves as his or her own control and provides information about the risk period and control period, the bias caused by individual confounding variables can be well controlled.

We conducted a 1:4 two-way case-crossover study design to estimate the short-term association between daily air pollutants exposure and the hospitalization risk for respiratory disease among young children aged < 2 years old. For each subject, the case day was defined as the day of hospital admission, and the control days were 7 and 14 days before and after the case day to eliminate the potential confounding effects of long-term trends, seasonality and week.

### Air pollution and meteorological data

The air pollution data of Wuhan during the study period were obtained from the Wuhan Environmental Protection Bureau. We downloaded hourly air pollutants concentrations from 18 air quality monitoring stations distributed in 13 districts of Wuhan and calculated the daily and annual concentrations for each station. Finally, particulate matter with an aerodynamic diameter less than 2.5 μm (PM_2.5_), sulfur dioxide (SO_2_), nitrogen dioxide (NO_2_) and ozone (O_3_) were included in this study. Climate data during this study period collected from the China Meteorological Data Network included the average daily temperature and relative humidity.

### Individual assessment of exposure to air pollutants

PM_2.5_ exposure assessment was based on the land use regression model (LUR model) with a spatial resolution of 1 × 1 km. In the process of modeling, we considered geographic predictor variables, including the types of land use, the length of roads, the nearest distance between the station and the road, the number of industrial sources, population density, and digital elevation. By setting up a multi-ring buffer, the relevant elements around the monitoring site were extracted to generate several variables. Multiple linear regression models were adopted to fit these variables with the average PM_2.5_ concentrations at the sites between the years 2017 and 2018 (*R*^2^ = 0.871). We divided the study area into a 1 × 1 km uniform grid and extracted the corresponding independent variables of grid points. PM_2.5_ concentrations were calculated by the established model. Kriging interpolation was used to generate a spatial distribution simulation charts of annual-mean PM_2.5_ concentrations in Wuhan (Fig. 1). After geocoding the exact residential address of patients, daily individual PM_2.5_ exposure concentration was calculated, following the method described in previous studies [20].

Assessment of individual exposure to gaseous pollutants using LUR model was not satisfactory (*R*^2^ < 0.60). Therefore, inverse distance weighting (IDW) was used to more accurately assess the exposure of NO_2_ (*R*^2^ = 0.73), SO_2_ (*R*^2^ = 0.65), and O_3_ (*R*^2^ = 0.72) [6, 21].

### Statistical analysis

We performed a conditional logistic regression analysis to separately examine the short-term effects of PM_2.5_, NO_2_, SO_2_ and O_3_ exposures on hospital admissions for total respiratory disease, as well as for pneumonia and acute respiratory infection (ARI). To adjust for the nonlinear confounding effects of both temperature and relative humidity, we added a natural cubic spline (NCS) function with 3 degrees of freedom (df) for them in the main analytic models. We separately included four air pollutants as continuous terms in the regression models and estimated the odds ratios (ORs) and their 95% confidence intervals (CIs), which corresponds to the increase in odds of hospital admissions associated with a 10 μg/m^3^ increase in exposure to each pollutant. Furthermore, taking into account the lag effect of air pollutant exposures, concentration of each pollutant at single-lag (lag 0 to lag 7) and several days’ moving-average (lag 0–1 to lag 0–7) before admissions were analyzed independently in the model.

In addition, several sensitivity analyses were performed to check the effects of patients’ gender (male, female), age (1–3 months, 4–12 months and 13–24 months) and home addresses region (central urban area and far urban area) for the models. To examine the effect of seasons on pollutants-hospitalization associations, we divided the study period into warm and cold months according to date for admission. Warm season was defined from May to October, while cold season was from November to April of the following year.

As the previous study, linearity for exposure–response curves between each pollutant and the risks of hospitalization were further checked by smoothing the air pollutants terms using NCS function (with 3 df) instead of including linear terms [22].

Statistical analyses were performed using R (version 3.6.0). All tests were two-sided, and *P* < 0.05 was considered statistically significant.

## Results

We identified 62,425 hospital admissions with a primary diagnosis of respiratory diseases with an average age of 0.89 ± 0.51 years during the study period, in which the number of pneumonia admissions were 36,295, accounting for more than 58%. Among the three diseases, the majority of the hospital admissions were for young children between 3 and 12 months of age (> 45%), and 60%of patients were males. The number of admissions for respiratory diseases and pneumonia in warm season was lower than in cold season except that for ARI. As to the region distribution for hospital admissions, we found that there were slightly fewer cases in central urban area than in far urban area (Table 1).

**Table 1:** Basic characteristics of hospital admission cases for respiratory diseases in Wuhan, China, 2017–2018

Population characteristic	Total respiratory diseases	Pneumonia	Acute respiratory infection
Participants, *n*	62,425	36,295	25,076
Age, y, mean ± SD	0.89 ± 0.51	0.86 ± 0.51	0.92 ± 0.49
Age group, mon, *n* (%)			
1–3	7115 (11.4)	5097 (14.0)	1881 (7.5)
3–12	29,852 (47.8)	16,781 (46.2)	12,549 (50)
12–24	25,458 (40.8)	14,417 (39.7)	10,646 (42.5)
Sex, *n* (%)			
Male	39,649 (63.5)	23,125 (63.7)	15,867 (63.3)
Female	22,776 (34.5)	13,170 (36.3)	9209 (36.7)
Season at admission, *n* (%)			
Warm	29,017 (46.5)	15,283 (42.1)	13,163 (52.5)
Cold	33,408 (53.5)	21,012 (57.9)	11,913 (47.5)
Region, *n* (%)			
Central urban area	29,503 (47.3)	16,843 (46.4)	12,176 (48.6)
Far urban area	32,922 (52.7)	19,452 (53.6)	12,900 (51.4)

*SD* standard deviation

During the study period the daily number of hospitalized patients with respiratory disease varied from 47 to 157 (daily mean, 96.1), of which pneumonia accounted for more than half (Table 2). The average concentrations of PM_2.5_, NO_2_, SO_2_ and O_3_ were 48.7 ± 29.3 μg/m^3^, 44.9 ± 19.2 μg/m^3^, 28.7 ± 14.4 μg/m^3^ and 138.3 ± 57.8 μg/m^3^, respectively. The levels of all air pollutants were much higher than the WHO air quality guideline values. Additionally, the concentrations of air pollutants were higher in winter and lower in summer except that of O_3_ (Fig. S2). Annual mean temperature and humidity of Wuhan were 17.5 ± 9.3 degrees Celsius and 79.4 ± 10.3 percent, respectively (Table 2).

**Table 2:** Summary characteristics of ambient air pollutants and daily hospitalization data in Wuhan, China, 2017–2018

Variables	Mean ± SD	Min	P25	Median	P75	Max
Pollution						
PM_2.5_ (μg/m^3^)	48.7 ± 29.3	7.23	27.71	42.28	61.47	211.5
NO_2_ (μg/m^3^)	44.9 ± 19.2	12.84	29.58	41.58	57.14	104.75
SO_2_ (μg/m^3^)	28.7 ± 14.4	8.83	13.01	24.87	35.67	89.83
O_3_ (μg/m^3^)	138.3 ± 57.8	11.91	92.62	140.2	177.09	376.33
Weather conditions						
Temperature (°C)	17.5 ± 9.3	− 3.8	9.48	18.1	25.8	33.9
Humidity (%)	79.4 ± 10.3	47	72	80	87	100
Daily case (*n*)						
RD	96.1 ± 16.3	47	75	85	95	157
Pneumonia	50.1 ± 13.8	22	40	49	58	104
ARI	34.6 ± 7.9	13	29	34	40	64

*PM*_*2.5*_ particles with aerodynamic diameter ≤ 2.5 μm; *NO*_*2*_ nitrogen dioxide; *SO*_*2*_ sulfur dioxide; *O*_*3*_ ozone; *RD* total respiratory disease; *ARI* acute respiratory infection; *SD* standard deviation; *Min* minimal value; *P25* lower quartile; *P75* upper quartile; Max, maximum value

To explore the relationship between air pollution and hospital admissions for respiratory disease, we performed a series of conditional logistic regression analyses. We observed that a 10 μg/m^3^ increase in exposure to PM_2.5_, NO_2_, and O_3_ was significantly associated with the increased odds of hospital admissions for respiratory diseases and pneumonia, but not with the OR for ARL (Table S1–S4). The lag days and OR values with the strongest association to hospitalization for each pollutant were various (Table 3). Overall, the effects of PM_2.5_ and NO_2_ on admissions were stronger than those of other pollutants. The risk of hospitalization for pneumonia was higher than that for respiratory diseases. In a single-day lag analysis, NO_2_ had the strongest effect on hospitalization at lag 0 day. ORs per 10 μg/m^3^ increase of NO_2_ were 1.0090 (95% CI 1.0043–1.0140) for respiratory diseases, and 1.0120 (95% CI 1.0059–1.0180) for pneumonia. Interestingly, in a moving-average lag analysis, the contribution of PM_2.5_ to hospitalization was higher. ORs per 10 μg/m^3^ increase of PM_2.5_ at lag 0–7 days were 1.0108 (95% CI 1.0044–1.0170) for respiratory diseases, and 1.0179 (95% CI 1.0097–1.0260) for pneumonia.

**Table 3:** Odds ratio (with 95% CIs) of hospitalization for respiratory diseases, pneumonia and ARI at various exposure days associated with per 10 μg/m^3^ increase in exposure to PM_2.5_, NO_2_, SO_2_ and O_3_

Disease	Pollutant	Single-lag		Moving-average lag	
		Day	OR (95% CIs)	Day	OR (95% CIs)
RD	PM_2.5_	0	1.0063 (1.0030–1.0100)	0–7	1.0108 (1.0044–1.0170)
	NO_2_	0	1.0090 (1.0043–1.0140)	0–1	1.0090 (1.0040–1.0140)
	SO_2_	7	1.0044 (0.9985–1.0100)	0–7	1.0018 (0.9926–1.0110)
	O_3_	5	1.0024 (1.0010–1.0040)	0–7	1.0022 (0.9990–1.0050)
Pneumonia	PM_2.5_	0	1.0105 (1.0057–1.0150)	0–7	1.0179 (1.0097–1.0260)
	NO_2_	0	1.0120 (1.0059–1.0180)	0–7	1.0131 (1.0042–1.0220)
	SO_2_	4	1.0084 (1.0006–1.0160)	0–7	1.0083 (0.9961–1.0210)
	O_3_	5	1.0033 (1.0008–1.0060)	0–7	1.0037 (0.9994–1.0080)
ARI	PM_2.5_	6	1.0055 (0.9992–1.0120)	0–7	0.9998 (0.9893–1.0100)
	NO_2_	0	1.0050 (0.9971–1.0120)	0–1	1.0050 (0.9971–1.0140)
	SO_2_	7	1.0007 (0.9912–1.0100)	0–1	0.9965 (0.9852–1.0080)
	O_3_	6	1.0030 (1.0002–1.0060)	0–7	1.0011 (0.9962–1.0060)

Day, exposure days with the largest effect in the single-day lag analysis and moving-average lag analysis

*PM*_*2.5*_ particles with aerodynamic diameter ≤ 2.5 μm; *NO*_*2*_ nitrogen dioxide; *SO*_*2*_ sulfur dioxide; *O*_*3*_ ozone; *RD* total respiratory disease; *ARI* acute respiratory infection; *OR* odds ratios; *CIs* confidence intervals; All analysis was controlled for temperature and humidity

**Fig. 2:**
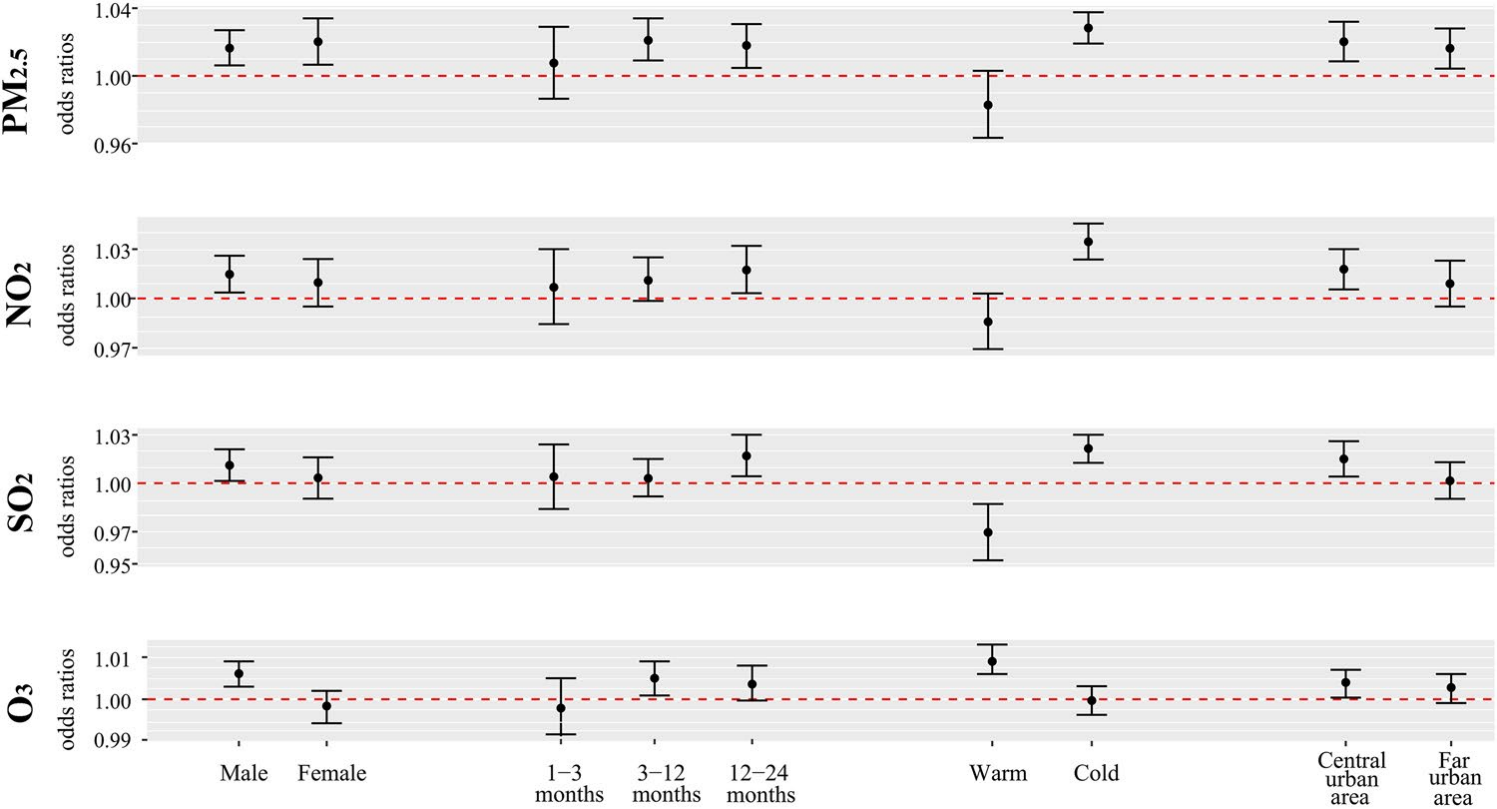
Odds ratios (with 95% CIs) of hospitalization for pneumonia among subgroups stratified by sex, age, season and region associated with per 10 μg/m^3^ increase in exposure to PM_2.5_, NO_2_, SO_2_ and O_3_. All analysis was controlled for temperature and humidity. CIs, confidence intervals; PM_2.5_, particles with aerodynamic diameter ≤ 2.5 μm; NO_2_, nitrogen dioxide; SO_2_, sulfur dioxide; O_3_, ozone

Subgroup analysis showed that PM_2.5_ had significant and comparable effects on children of both genders, with ORs of 1.0164 (95% CI 1.0062–1.0270) for males and 1.0202 (95% CI 1.0066–1.0340) for females (Fig. 2). However, the risks of exposure to gaseous pollutants (NO_2_, SO_2_ and O_3_) have been observed only in males. Air pollutants associated to the risks of hospitalization differed substantially among month age groups. In the elder month age group, the hospitalization risk had an increasing trend. In the seasonal subgroup analysis, PM_2.5_, NO_2_ and SO_2_ were strongly associated with hospitalization in the cold season, with ORs of 1.0283 (95% CI 1.0191–1.0376), 1.0345 (95% CI 1.0237–1.0456) and 1.0215 (95% CI 1.0126–1.0300), whereas the effect of O_3_ was stronger in the warm season with OR of 1.009 (95% CI 1.0060–1.0130). In addition, the risks of hospitalization for exposure to each pollutant were significantly higher in the central urban areas than in the far urban areas.

**Fig. 3:**
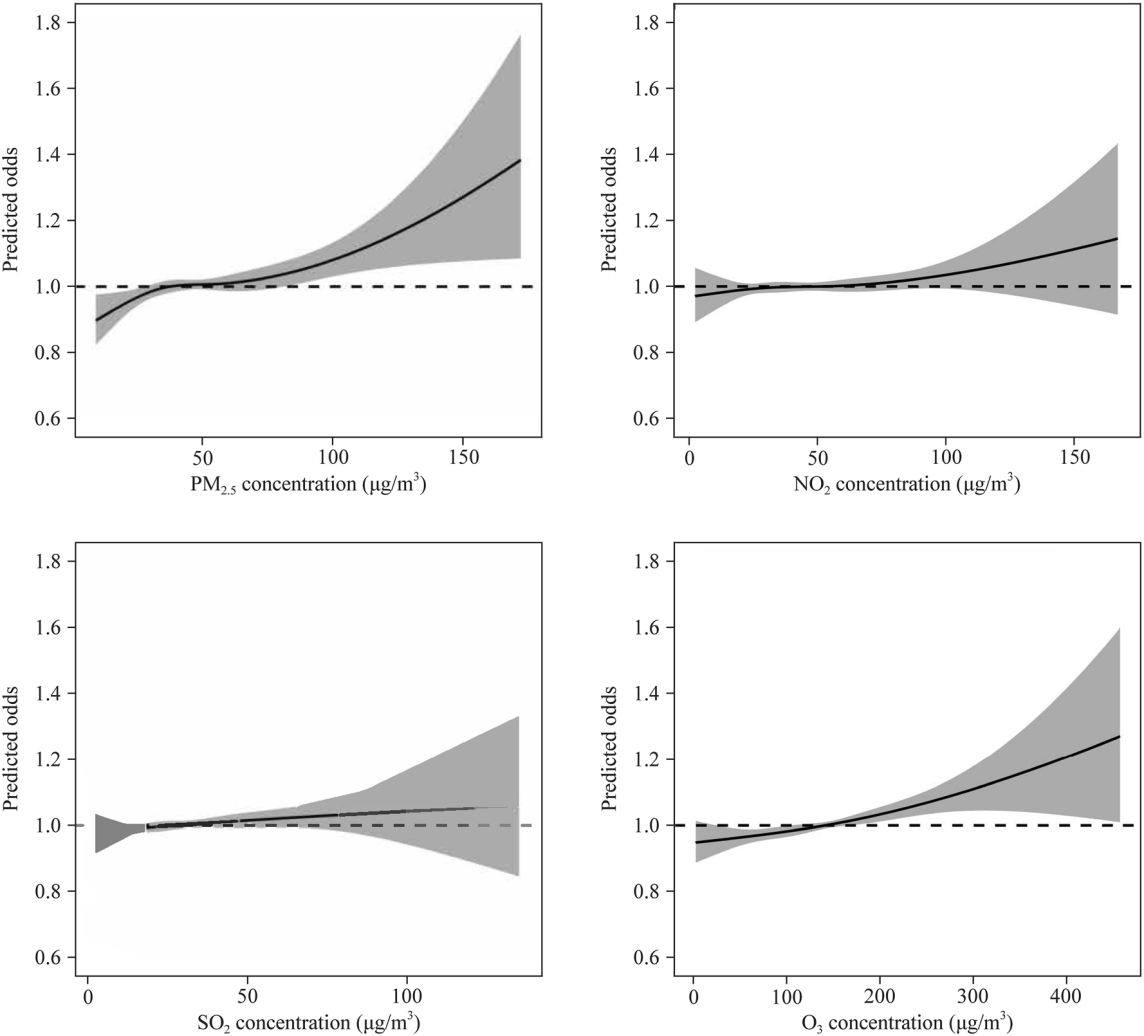
Concentration–response curves (smoothing by NCS function with *df* = 3) between air pollutants and risks of hospital admission for pneumonia. The exposure days of each pollutant were selected as the days with the strongest effect in the lag analysis. All analysis was controlled for temperature and humidity. The solid line represents the predicted odds of hospital admission and the shadow represents the 95% CIs. NCS, natural cubic spline; CIs, confidence intervals; PM_2.5_, particles with aerodynamic diameter ≤ 2.5 μm; *NO*_*2*_ nitrogen dioxide; *SO*_*2*_ sulfur dioxide; *O*_*3*_ ozone

The dose–response relationship between gaseous pollutants and hospitalization for pneumonia showed a similar pattern, which was mainly linear, without obvious threshold effect (Fig. 3). But in the case of PM_2.5_, the relationship fluctuated, with the stronger response at low (< 35 μg/m^3^) and high concentrations (> 60 μg/m^3^).

## Discussion

In this case crossover study, we estimated the short-term impact of air pollution on hospital admissions on respiratory diseases among young children aged < 2 years based on an entire children population of a megalopolis and the precise individual assessment of pollution exposures. Our analysis showed that exposure to various air pollutants was obviously associated with a higher risk of hospital admissions for total respiratory disease and pneumonia. The associations between air pollution and the risks of hospital admissions differed in subgroup analysis including gender, age and season. The results provided sufficient evidence of the harmful effects of air pollution on children's respiratory system in Wuhan; and the results may help public agencies to develop strategies for air pollution control and disease prevention and to promote local maternal and child health development.

Air pollutants showed significant effects on respiratory diseases, especially on pneumonia, and our findings were consistent with those of other studies [8, 23]. However, ORs of hospitalization risk for pneumonia were 1.0179 (95% CI 1.0097–1.0260), 1.0131 (95% CI 1.0042–1.0220) for PM_2.5_ and NO_2_ at lag 0–7 days in this study, which were lower than that in other similar studies. A study in Shenzhen City of China found that the risk of hospital admission for respiratory diseases was 1.06 (95% CI 1.02–1.10) in exposure to PM_2.5_ [23]. Another meta-analysis showed the increased risks were 1.8% (0.5–3.1%) for PM_2.5_, 1.4% (0.4–2.4%) for NO_2_, 2.9% (0.4–5.3%) for SO_2_ and 1.7% (0.5–2.8%) for O_3_ [3]. The studies based on the whole population of a megalopolis may generally have the lower parameter estimates than the studies based on the sample population, indicating that selection bias may lead to an overestimation of hospitalization risk [4, 7, 24, 25]. Additionally, the concentrations of air pollutants in Wuhan were higher than those in other study areas and also were much higher than the WHO guideline values. Thus, long-term high level exposure may result in a low sensitivity of the population to an increase concentration of short-term exposure to air pollutants [26].

In this study, the health effects of PM_2.5_ and NO_2_ were estimated to be higher than those of other air pollutants. PM_2.5_ has complex components, small particle size and large specific surface area, which can absorb toxic substances in the air and can lead to diseases by promoting oxidative stress, causing inflammation and weakening host immune defense ability [27]. NO_2_ is a primary pollutant of traffic emission and one of main components of urban air pollution, which mainly invades deep bronchioles and alveoli of respiratory tract, and can strongly irritate lung tissue [28]. In comparison with PM_2.5_, the effect of NO_2_ was stronger than that of PM_2.5_ in the single-day lag analysis, but was weaker in the moving-average lag analysis. This may be attributed to the different health impact mechanisms of the two pollutants, suggesting that the adverse health effects of PM_2.5_ are milder but more likely to be accumulated.

A single-day lag analysis showed that the hospitalization risk for respiratory diseases and pneumonia was highest within 24 hours after acute exposure to PM_2.5_ and NO_2_, but the strongest adverse effects of SO_2_ and O_3_ occurred three to four days after exposure (Table 3). The results suggest that the effects of PM_2.5_ and NO_2_ on children's respiratory diseases may be more rapid and invasive. A time series study in Xi’an of China observed the strongest correlation between PM_2.5_ and respiratory mortality at lag 0 day. Statistically significant associations also were observed for SO_2_ at lag 4 day [29]. In Poland, a study found a statistically significant increase of hospitalizations for respiratory disease (between 0.9 and 4.5% increase per 10 units of pollutant increase) after peaks of PM_2.5_ concentrations, with a typical time lag between the pollutant peak and the event of 2–6 days [30]. The above findings make us realize that the time-effect relationship after exposure to air pollutants may be very complicated and needs further investigations. The variation of the strongest impact days of different air pollutants may be attributed to a combination of complex factors, such as the different types of diseases, individual behavior patterns, differences in air pollution components and concentrations, and influence mechanisms. In the moving-average lag analysis, the association between air pollution exposure and the risk of hospital admissions was further strengthened. Considering short-term cumulative effects, the moving average lag may be a better measure of exposure time. The results in this study provide solid evidence of the influence patterns and the importance of exposure time to air pollution.

In environmental epidemiological studies, the gender difference in the associations between air pollution and health effect has been widely concerned, but no consistent conclusions have been reached so far [31]. During the study period, there were more male children admitted to hospital for respiratory disease than female children. We observed the significant effects of PM_2.5_ on hospital admissions for pneumonia among both genders, but NO_2_, SO_2_ and O_3_ have effects on male children only (Fig. 2), suggesting that males were more sensitive to the increases of exposure to air pollutants. Our results were consistent with those of several previous studies [29, 32]. A study described that the link between air pollution and respiratory problems was stronger in boys among young children; but older childhood cohorts suggest the opposite [31]. The age-related trends may be linked to sex-differing lung function growth rates and differences in airway function at birth, which suggest lower respiratory volumes and greater airway resistance among boys. At elder ages, gendered activities may shape pollution response [24].

We examined seasonal differences in the effects of ambient air pollution on young children. In this study, the association between PM_2.5_, NO_2_, and SO_2_ and hospitalization for pneumonia in children was observed in the cold season. Conversely, O_3_ was associated with hospitalization for pneumonia in the warm season. These findings were consistent with previous studies [33, 34]. The difference in season specific effect estimates may reflect seasonal variations of the concentration, compositions and toxicity of different air pollutants [35]. In Wuhan the concentrations of air pollutants in the cold season are higher than those in the cold season except O_3_. A dry climate with little rain tends to result in the accumulation of pollutants, and low wind speed also limits the spread of pollutants. In the meantime, viruses are more likely to attach to particles at lower temperatures [36]. In the warm season, strong sunlight and high temperatures accelerate photochemical reactions, making O_3_ the primary pollutant affecting air quality and residents' health [37]. Estimates of air pollution effects are also different in central and far urban areas, which may be related to regional population behavior patterns, vegetation greening, pollutants concentration and composition differences [20, 23]. These results suggest that we have different protection priorities for sensitive people in different seasons and regions.

There were a number of limitations in this study. First, we included hospital admissions from 54 municipal hospitals in Wuhan, but we may have ignored admissions from small hospitals located in communities. Therefore, the admissions we included were likely to be more severe, leading to an underestimation of the impact of air pollution. Secondly, an interesting finding in our study was that children's exposure to air pollution was associated with a higher risk of hospital admission with an increasing age trend. A potential explanation of this higher risk in elder month age is the protective effect of breastfeeding against respiratory infections [38]. In China, due to a well-done propaganda of breastfeeding, the rate of breastfeeding is relatively high for young children. However, lacking of data on breastfeeding, it is not possible to evaluate this effect quantitatively, and further studies are needed in the future. Finally, although LUR and IDW models were used to fit spatial and temporal differences in air pollution, there was a lack of direct individual exposure and indoor air pollution exposure measurements for patients, which might lead to misclassification of exposure concentration.

In conclusion, the present study confirms that exposure to ambient air pollution is associated with a higher risk of hospital admission for total respiratory disease and pneumonia among young children aged < 2 years in Wuhan. The male and elder month children are more sensitive to the increases of exposure to air pollutants. In addition, a significant stronger association between air pollutants other than O_3_ and the risk of hospital admission for respiratory diseases is observed during cold seasons. These findings may contribute to regional public health policies to reduce the burden of childhood disease.

## Supplementary Information

Below is the link to the electronic supplementary material.

Supplementary file1 (DOCX 810 KB)

## Data Availability

The data that support the findings of this study are available from Wuhan Information Center of Health and Family Planning but restrictions apply to the availability of these data, which were used under license for the current study, and so are not publicly available. Data are however available from the authors upon reasonable request and with permission of Wuhan Information Center of Health and Family Planning.
