# Supplementary material for: Short-term effects of air pollution on respiratory diseases among young children in Wuhan city, China
Source: World J Pediatr. 2022 Mar 25;18(5):333–42. doi: 10.1007/s12519-022-00533-5 (PMC9042971; doi:10.1007/s12519-022-00533-5)
Supplement: Supplementary file 1 — Supplementary file1 (DOCX 810 KB) [file 12519_2022_533_MOESM1_ESM.docx]

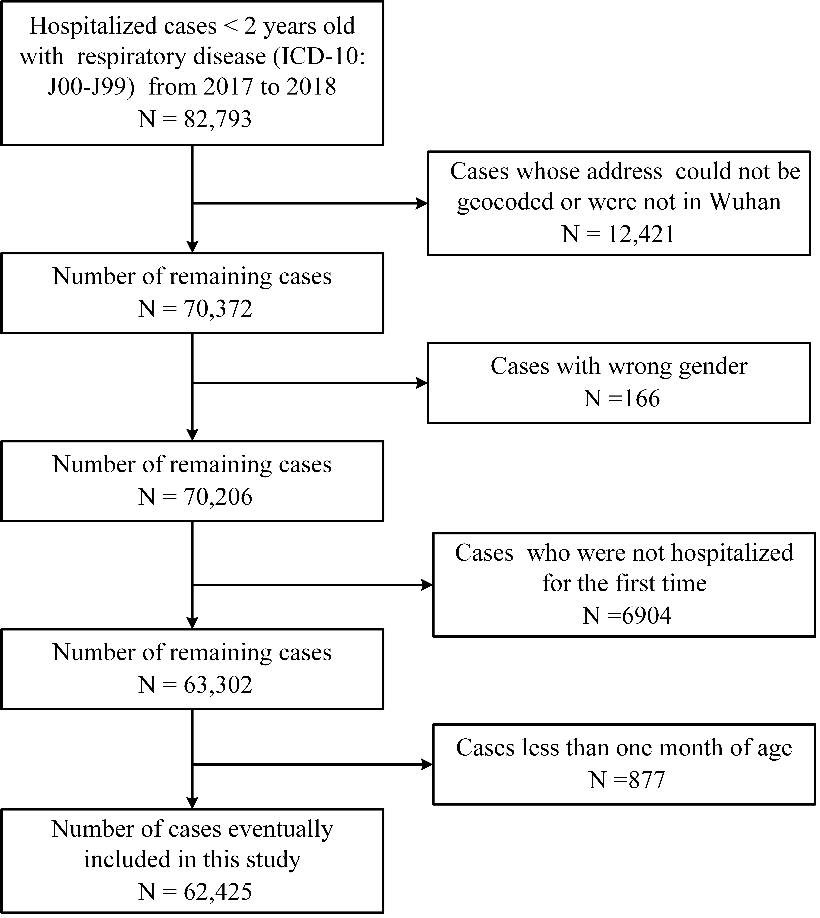


**Fig. S1.** Flowchart of cases recruitment in this study.


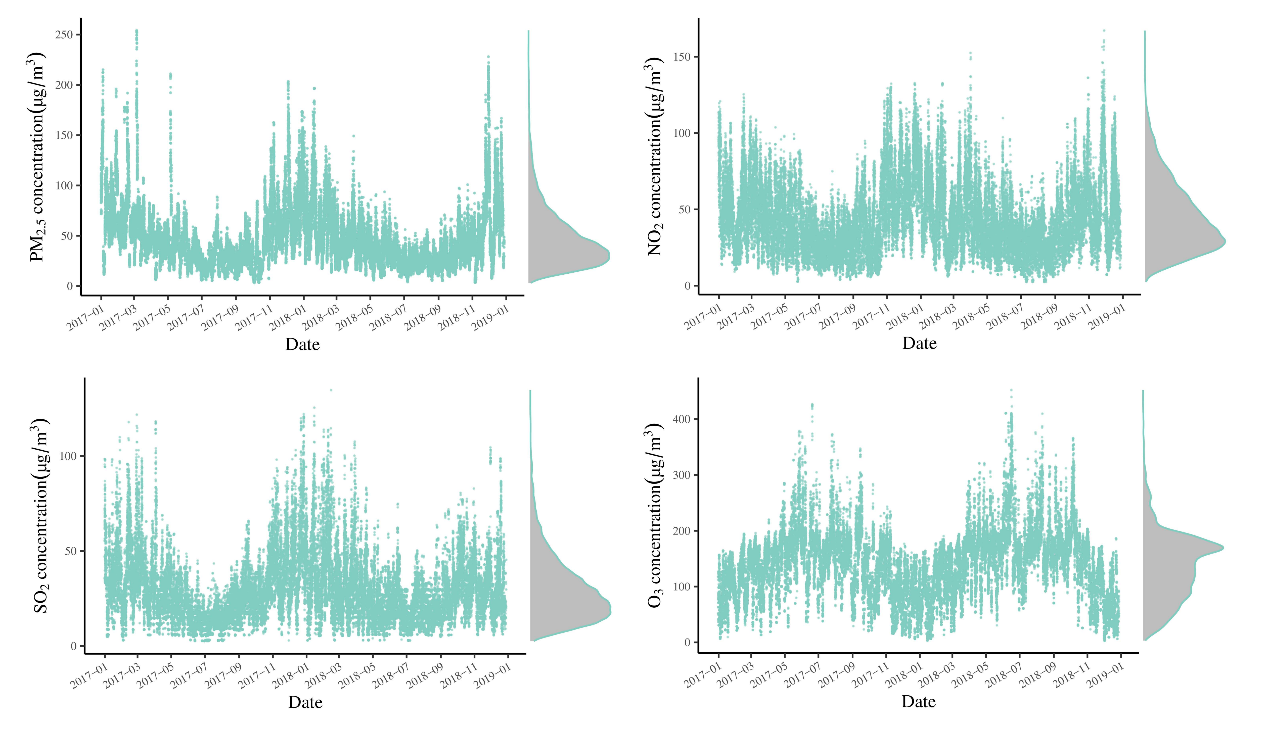


**Fig. S2.** Distribution of individual’s air pollution exposure on the day of admission. The points present the air pollutants concentration of individuals. The density plot on the right marginal of y-axis visualizes the distribution of the number of subjects over the air pollutants concentration.

**Table S1** Odds ratios (with 95% CIs) of hospitalization for TRD, pneumonia and ARI at various exposure days associated with per 10μg/m^3^ increase in exposure to PM_2.5_.

| Lag days | Total respiratory diseases | Pneumonia | Acute respiratory infection |
| --- | --- | --- | --- |
| Lag 0 | 1.0063 (1.0030-1.0100) | 1.0105 (1.0057-1.0150) | 0.9995 (0.9932-1.0060) |
| Lag 1 | 1.0045 (1.0010-1.0080) | 1.0082 (1.0034-1.0130) | 0.9986 (0.9923-1.0050) |
| Lag 2 | 1.0042 (1.0000-1.0080) | 1.0079 (1.0031-1.0130) | 0.9987 (0.9925-1.0050) |
| Lag 3 | 1.0051 (1.0010-1.0090) | 1.0103 (1.0055-1.0150) | 0.9970 (0.9908-1.0030) |
| Lag 4 | 1.0040 (1.0000-1.0080) | 1.0062 (1.0014-1.0110) | 1.0010 (0.9951-1.0080) |
| Lag 5 | 1.0046 (1.0008-1.0080) | 1.0058 (1.0010-1.0110) | 1.0025 (0.9962-1.0090) |
| Lag 6 | 1.0048 (1.0010-1.0090) | 1.0040 (0.9991-1.0090) | 1.0055 (0.9992-1.0120) |
| Lag 7 | 1.0037 (0.9999-1.0080) | 1.0033 (0.9985-1.0080) | 1.0044 (0.9981-1.0110) |
|  |  |  |  |
| Lag 0-1 | 1.0062 (1.0020-1.0100) | 1.0112 (1.0058-1.0170) | 0.9983 (0.9913-1.0050) |
| Lag 0-2 | 1.0065 (1.0020-1.0110) | 1.0124 (1.0065-1.0180) | 0.9975 (0.9899-1.0050) |
| Lag 0-3 | 1.0073 (1.0020-1.0120) | 1.0149 (1.0086-1.0210) | 0.9955 (0.9873-1.0040) |
| Lag 0-4 | 1.0080 (1.0030-1.0130) | 1.0158 (1.0090-1.0230) | 0.9962 (0.9874-1.0050) |
| Lag 0-5 | 1.0090 (1.0033-1.0150) | 1.0169 (1.0096-1.0240) | 0.9969 (0.9875-1.0060) |
| Lag 0-6 | 1.0101 (1.0040-1.0160) | 1.0175 (1.0098-1.0250) | 0.9986 (0.9887-1.0090) |
| Lag 0-7 | 1.0108 (1.0044-1.0170) | 1.0179 (1.0097-1.0260) | 0.9998 (0.9893-1.0100) |

Legends: lag days, exposure days in the single-day lag analysis and moving-average lag analysis. All analysis was controlled for temperature and humidity.

**Table S2** Odds ratios (with 95% CIs) of hospitalization for TRD, pneumonia and ARI at various exposure days associated with per 10μg/m^3^ increase in exposure to NO_2_.

| Lag days | Total respiratory diseases | Pneumonia | Acute respiratory infection |
| --- | --- | --- | --- |
| Lag 0 | 1.0090 (1.0043-1.0140) | 1.0120 (1.0059-1.0180) | 1.0050 (0.9971-1.0120) |
| Lag 1 | 1.0070 (1.0025-1.0120) | 1.0081 (1.0021-1.0140) | 1.0050 (0.9978-1.0130) |
| Lag 2 | 1.0040 (0.9994-1.0090) | 1.0046 (0.9987-1.0110) | 1.0030 (0.9951-1.0100) |
| Lag 3 | 1.0040 (0.9998-1.0090) | 1.0057 (0.9999-1.0120) | 1.0010 (0.9936-1.0090) |
| Lag 4 | 1.0034 (0.9988-1.0080) | 1.0080 (1.0022-1.0140) | 0.9959 (0.9884-1.0030) |
| Lag 5 | 1.0013 (0.9968-1.0060) | 1.0042 (0.9985-1.0100) | 0.9964 (0.9890-1.0040) |
| Lag 6 | 1.0016 (0.9971-1.0060) | 1.0037 (0.9979-1.0090) | 0.9970 (0.9896-1.0050) |
| Lag 7 | 1.0029 (0.9984-1.0070) | 1.0039 (0.9982-1.0100) | 1.0006 (0.9931-1.0080) |
|  |  |  |  |
| Lag 0-1 | 1.0090 (1.0040-1.0140) | 1.0110 (1.0049-1.0180) | 1.0050 (0.9971-1.0140) |
| Lag 0-2 | 1.0090 (1.0031-1.0140) | 1.0110 (1.0036-1.0180) | 1.0050 (0.9961-1.0140) |
| Lag 0-3 | 1.0090 (1.0027-1.0140) | 1.0110 (1.0035-1.0180) | 1.0040 (0.9944-1.0140) |
| Lag 0-4 | 1.0090 (1.0026-1.0150) | 1.0120 (1.0047-1.0200) | 1.0020 (0.9921-1.0120) |
| Lag 0-5 | 1.0080 (1.0017-1.0150) | 1.0126 (1.0044-1.0210) | 1.0002 (0.9897-1.0110) |
| Lag 0-6 | 1.0080 (1.0013-1.0150) | 1.0129 (1.0044-1.0220) | 0.9990 (0.9881-1.0100) |
| Lag 0-7 | 1.0084 (1.0014-1.0150) | 1.0131 (1.0042-1.0220) | 0.9994 (0.9880-1.0110) |

Legends: lag days, exposure days in the single-day lag analysis and moving-average lag analysis. All analysis was controlled for temperature and humidity.

**Table S3** Odds ratios (with 95% CIs) of hospitalization for TRD, pneumonia and ARI at various exposure days associated with per 10μg/m^3^ increase in exposure to SO_2_.

| Lag days | Total respiratory diseases | Pneumonia | Acute respiratory infection |
| --- | --- | --- | --- |
| Lag 0 | 0.9989 (0.9924-1.006) | 0.9989 (0.9903-1.0080) | 0.9976 (0.9872-1.0080) |
| Lag 1 | 0.9996 (0.9933-1.0060) | 0.9999 (0.9918-1.0080) | 0.9985 (0.9886-1.0080) |
| Lag 2 | 1.0030 (0.9969-1.0090) | 1.0067 (0.9988-1.0150) | 0.9978 (0.9882-1.0070) |
| Lag 3 | 0.9999 (0.9939-1.0060) | 1.0030 (0.9952-1.0110) | 0.9952 (0.9857-1.0050) |
| Lag 4 | 1.0030 (0.9971-1.0090) | 1.0084 (1.0006-1.0160) | 0.9957 (0.9863-1.0050) |
| Lag 5 | 1.0009 (0.9950-1.0070) | 1.0019 (0.9941-1.0100) | 0.9998 (0.9903-1.0090) |
| Lag 6 | 1.0038 (0.9978-1.0100) | 1.0075 (0.9997-1.0150) | 0.9985 (0.9890-1.0080) |
| Lag 7 | 1.0044 (0.9985-1.0100) | 1.0072 (0.9994-1.0150) | 1.0007 (0.9912-1.0100) |
|  |  |  |  |
| Lag 0-1 | 0.9985 (0.9914-1.0060) | 0.9990 (0.9897-1.0080) | 0.9965 (0.9852-1.0080) |
| Lag 0-2 | 0.9999 (0.9924-1.0070) | 1.0023 (0.9924-1.0120) | 0.9956 (0.9838-1.0080) |
| Lag 0-3 | 0.9993 (0.9914-1.0070) | 1.0029 (0.9925-1.0130) | 0.9935 (0.9812-1.0060) |
| Lag 0-4 | 1.0001 (0.9919-1.0080) | 1.0050 (0.9945-1.0160) | 0.9923 (0.9795-1.0050) |
| Lag 0-5 | 0.9999 (0.9913-1.0080) | 1.0049 (0.9936-1.0160) | 0.9925 (0.9792-1.0060) |
| Lag 0-6 | 1.0008 (0.9919-1.0100) | 1.0067 (0.9950-1.0190) | 0.9922 (0.9785-1.0060) |
| Lag 0-7 | 1.0018 (0.9926-1.0110) | 1.0083 (0.9961-1.0210) | 0.9926 (0.9784-1.0070) |

Legends: lag days, exposure days in the single-day lag analysis and moving-average lag analysis. All analysis was controlled for temperature and humidity.

**Table S4** Odds ratios (with 95% CIs) of hospitalization for TRD, pneumonia and ARI at various exposure days associated with per 10μg/m^3^ increase in exposure to O_3_.

| Lag days | Total respiratory diseases | Pneumonia | Acute respiratory infection |
| --- | --- | --- | --- |
| Lag 0 | 1.0000 (0.9979-1.0020) | 1.0004 (0.9977-1.0030) | 0.9999 (0.9967-1.0030) |
| Lag 1 | 0.9989 (0.9969-1.0010) | 0.9987 (0.9960-1.0010) | 0.9991 (0.9960-1.0020) |
| Lag 2 | 0.9990 (0.9971-1.0010) | 0.9993 (0.9967-1.0020) | 0.9994 (0.9964-1.0020) |
| Lag 3 | 1.0010 (0.9987-1.0020) | 1.0019 (0.9994-1.0040) | 0.9995 (0.9966-1.0020) |
| Lag 4 | 1.0010 (0.9990-1.0030) | 1.0019 (0.9994-1.0040) | 0.9999 (0.9971-1.0030) |
| Lag 5 | 1.0024 (1.0010-1.0040) | 1.0033 (1.0008-1.0060) | 1.0014 (0.9985-1.0040) |
| Lag 6 | 1.0023 (1.0004-1.0040) | 1.0019 (0.9994-1.0040) | 1.0030 (1.0002-1.0060) |
| Lag 7 | 1.0015 (0.9996-1.0030) | 1.0022 (0.9997-1.0050) | 1.0005 (0.9977-1.0030) |
|  |  |  |  |
| Lag 0-1 | 0.9992 (0.9968-1.0020) | 0.9993 (0.9961-1.0020) | 0.9993 (0.9956-1.0030) |
| Lag 0-2 | 0.9988 (0.9962-1.0010) | 0.9991 (0.9956-1.0030) | 0.9991 (0.9952-1.0030) |
| Lag 0-3 | 0.9993 (0.9966-1.0020) | 1.0003 (0.9966-1.0040) | 0.9990 (0.9948-1.0030) |
| Lag 0-4 | 0.9998 (0.9969-1.0030) | 1.0011 (0.9972-1.0050) | 0.9990 (0.9946-1.0030) |
| Lag 0-5 | 1.0008 (0.9978-1.0040) | 1.0020 (0.9983-1.0060) | 0.9997 (0.9951-1.0040) |
| Lag 0-6 | 1.0016 (0.9985-1.0050) | 1.0029 (0.9987-1.0070) | 1.0010 (0.9962-1.0060) |
| Lag 0-7 | 1.0022 (0.9990-1.0050) | 1.0037 (0.9994-1.0080) | 1.0011 (0.9962-1.0060) |

Legends: lag days, exposure days in the single-day lag analysis and moving-average lag analysis. All analysis was controlled for temperature and humidity.
